# Nonidentifiability of the Source of Intrinsic Noise in Gene Expression from Single-Burst Data

**DOI:** 10.1371/journal.pcbi.1000192

**Published:** 2008-10-10

**Authors:** Piers J. Ingram, Michael P. H. Stumpf, Jaroslav Stark

**Affiliations:** 1 Department of Mathematics, Imperial College London, London, United Kingdom; 2 Centre for Integrative Systems Biology at Imperial College, Imperial College London, London, United Kingdom; 3 Theoretical Genomics Group, Centre for Bioinformatics, Division of Molecular Biosciences, Imperial College London, London, United Kingdom; University of California San Diego, United States of America

## Abstract

Over the last few years, experimental data on the fluctuations in gene activity between individual cells and within the same cell over time have confirmed that gene expression is a “noisy” process. This variation is in part due to the small number of molecules taking part in some of the key reactions that are involved in gene expression. One of the consequences of this is that protein production often occurs in bursts, each due to a single promoter or transcription factor binding event. Recently, the distribution of the number of proteins produced in such bursts has been experimentally measured, offering a unique opportunity to study the relative importance of different sources of noise in gene expression. Here, we provide a derivation of the theoretical probability distribution of these bursts for a wide variety of different models of gene expression. We show that there is a good fit between our theoretical distribution and that obtained from two different published experimental datasets. We then prove that, irrespective of the details of the model, the burst size distribution is always geometric and hence determined by a single parameter. Many different combinations of the biochemical rates for the constituent reactions of both transcription and translation will therefore lead to the same experimentally observed burst size distribution. It is thus impossible to identify different sources of fluctuations purely from protein burst size data or to use such data to estimate all of the model parameters. We explore methods of inferring these values when additional types of experimental data are available.

## Author Summary

Recent experimental data showing fluctuations in gene activity between individual cells and within the same cell over time confirm that gene expression is a “noisy” process. This variation is partly due to the small number of molecules involved in gene expression. One consequence is that protein production often occurs in bursts, each due to the binding of a single transcription factor. Recently, the distribution of the number of proteins produced in such bursts has been experimentally measured, offering a unique opportunity to study the relative importance of different sources of noise in gene expression. We derive the theoretical probability distribution of these bursts for a wide variety of gene expression models. We show a good fit between our theoretical distribution and experimental data and prove that, irrespective of the model details, the burst size distribution always has the same shape, determined by a single parameter. As different combinations of the reaction rates lead to the same observed distribution, it is impossible to estimate all kinetic parameters from protein burst size data. When additional data, such as protein equilibrium distributions, are available, these can be used to infer additional parameters. We present one approach to this, demonstrating its application to published data.

## Introduction

The regulation of gene activity is essential for the proper functioning of cells, which employ a variety of molecular mechanisms to control gene expression. Despite this, there is considerable variation in the precise number and timing of protein molecules that are produced for a given gene under any particular set of circumstances. This is because gene expression is fundamentally a “noisy” process, subject to a number of sources of randomness. Some of these are *intrinsic* to the biochemical reactions that comprise the transcription and translation of a particular gene [1],[2]. Several of the reactions involve very small numbers of molecules. There are only one or two copies of the DNA for the gene, and in its vicinity inside the cell there are likely to be only a few copies of the relevant transcription factors and of RNA polymerase. Similarly, for each mRNA molecule, the processes of ribosome binding and of mRNA degradation are typically highly stochastic.

Recent advances in experimental technology have shown that such single molecule effects can lead to protein production occurring in bursts of varying size, each due to a single transcription factor binding event [3],[4]. Other sources of variability are *extrinsic* to the specific reactions, and include fluctuations in relevant metabolites, polymerases, ribosomes, etc. [1],[2]. These will not be considered further here.

It is of considerable interest to determine the various contributions of such different sources of variability. Within the last few years, experimental techniques for addressing this question have increasingly become available. Elowitz et al. [1] observed fluctuations in the expression level of genes tagged both with cyan and yellow fluorescent proteins in monoclonal *Escherichia coli* cells under identical environmental conditions. Similar work was carried out by Raser and O'Shea [5] in the eukaryote *Saccharomyces cerevisiae*. Such dual-reporter experiments are able to distinguish between intrinsic and extrinsic sources of stochasticity. More recently, single molecule data has become available [6],[7], which monitors the expression of a gene a single protein at a time and provides the distribution of the sizes of bursts. It had been hoped that data of this kind would answer many of the remaining questions about the origin of noise in gene expression and in particular distinguish between the different contributions of transcription and translation to intrinsic noise.

Intuitively, one might expect that randomness due to transcription would play the more significant role than translation, since typically there will be more than one mRNA molecule, and the fluctuations due to translation from each of these might to some extent average out. To test this hypothesis and to put it on a quantitative basis, it is necessary to employ mathematical models of gene expression. These also provide a valuable tool for the analysis of experimental data, and in particular of the burst size distributions reported in the literature, e.g., [6],[7].

A great deal of work has gone into modelling gene expression in both prokaryotic and eukaryotic systems, with some of the earliest papers predicting fluctuations in mRNA and protein levels published 30 years ago [8],[9]. McAdams and Arkin [3] provided the first model of bursting at the translation level. They showed that the number of protein molecules produced by a single mRNA transcript is described well by a model which considers whether the next event is the production of a further protein, or the degradation of the mRNA molecule. Such competitive binding between ribosomes and RNase results in a geometric distribution for the protein number. Such an analysis can also be applied to transcription following the binding of a transcription factor to a gene and also results in a geometric distribution. The joint analysis of these two stochastic processes forms the basis of the present paper.

The integration of simple stochastic (Markov) models of transcription factor, RNA polymerase, ribosome and RNase binding leads to what is now widely regarded as the standard model of gene expression for prokaryotes [4]. The analysis of this model using a master equation allows the determination of the moments of the distribution of the number of protein molecules when the system is in steady state. Further analysis of this equilibrium distribution was carried out by Paulsson [10]–[12] who used the master equation and the fluctuation–dissipation theorem to obtain predictions about the mean and variances of molecule numbers and lifetimes and the contribution made by transcriptional and translational bursting. Other studies have been carried out by Höfer [13] who used a rapid-equilibrium approximation to compare mRNA levels for genes with one and two active alleles, and by Friedman et al. [14]. The drawback of these approaches is that the master equation that describes the temporal evolution of the probability distribution of protein (and mRNA) numbers is too complex to be solved analytically. Furthermore, the burst size distribution necessary for comparison with recent experimental data [6],[7] cannot be obtained directly from the master equation. Such difficulties with master equation based approaches are exacerbated in the case of more complex models of gene expression such as multi-step models that include intermediate stages such as the formation of DNA–RNA polymerase complexes, phosphorylation events, and mRNA–ribosome binding. Both deterministic and stochastic simulation studies of these models have been performed, e.g., [15] and [16], but none of these approaches have been useful for the analysis of burst size data.

In the present work we avoid the problems associated with the master equation approach, which are at least in part due to the explicit incorporation of time evolution. Instead, we ignore time and directly derive an expression for the burst size distribution by extending the analysis of [3]. In many ways this approach is similar to that used for the analysis of multi-stage queues [17]. The distribution of the number of mRNA molecules produced in a single burst is geometric and the distribution of the number of protein molecules produced by a single mRNA is also geometric [3]. The overall burst size distribution is therefore given by the compound distribution of two geometric distributions [17]. This can be readily computed using generating functions [17] and is itself *not* geometric. However, experimentally it is not possible to detect bursts that produce no protein molecules at all, and therefore the published data [6],[7] are in fact the relevant conditional distributions, assuming at least one protein molecule is produced in a burst. Surprisingly, it turns out that when we condition the compound distribution in this way, we again obtain a geometric distribution. This is determined by a single parameter, which we can derive in terms of physically meaningful constants such as binding and unbinding rates. This shows that different combinations of noise levels in the translation and transcription parts of the process can give the same overall burst size distribution. Mathematically, this means that the standard model of gene expression (described in detail below) is *nonidentifiable* [18],[19] from burst size data alone. This in turn implies that it is not possible to identify the relative contributions of translation and transcription to the burst size distribution of protein numbers only using this data.

We also show that our approach is applicable to a variety of more detailed models that incorporate additional steps to provide more realistic descriptions of expression [16]. These still yield a single parameter geometric conditional distribution. This shows that within the context of a very large class of models, experimental burst size data on its own cannot identify the relative contributions of different reactions to the overall noise level. However, by simulating the equilibrium distribution of protein numbers for different parameter combinations giving the same burst size distribution we demonstrate that a combination of burst size distribution and equilibrium distribution can discern different sources of noise. The difficulty with such an approach is that the determination of the equilibrium distribution requires the knowledge of two additional kinetic parameters: the transcription factor binding rate and the protein degradation rate. Estimates of these are not easy to obtain independently, so that we now have to estimate six unknown parameters from the combined burst size and equilibrium distribution data. Initial simulations (not shown here) suggested that it is difficult to do this reliably.

It is possible however, by using independent estimates of one of the parameters to reduce the parameter space from six to five dimensions. Using the relationship between the remaining parameters determined from the burst size distribution allows the elimination of a further parameter, leaving four kinetic parameters to be estimated from the equilibrium distribution. We show below that by using the Nelder-Mead algorithm to maximize the empirical likelihood, useful estimates of the four remaining parameters can be obtained. We carry out this process twice, first using independent measurements of the mRNA degradation rate and then of the protein half-life. In the first case we obtain unrealistically short estimates of the protein half-life, and in the second a considerably faster mRNA degradation. This suggests that when in the repressed state, mRNA may be degraded at a faster rate than when the gene is active.

In principle, this method can be applied to any gene where burst size and equilibrium distributions are available, providing a new approach to the estimation of parameters estimates for the ever more sophisticated models increasingly being used in computational biology.

## Methods

### The Standard Model of Gene Expression

**Figure 1:**
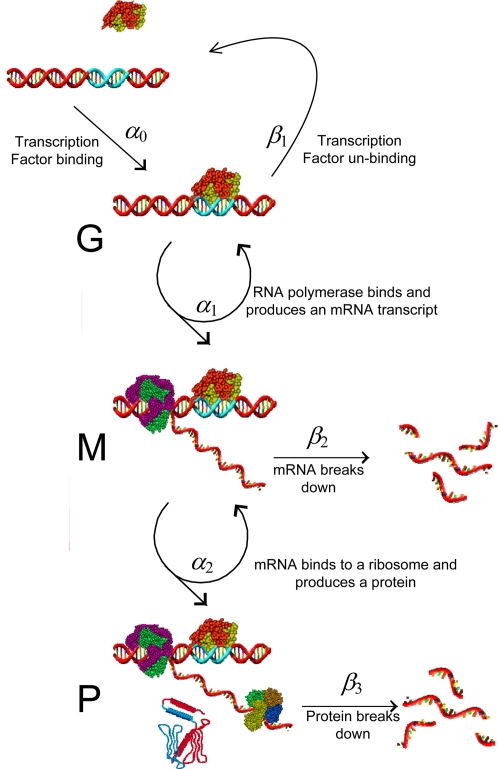
The standard gene expression model. An inactive sequence of DNA and a transcription factor bind to produce an active gene *G*. This produces mRNA, denoted by *M* at a rate *α*_1_, and in turn the mRNA produces protein at rate *α*_2_. Eventually, the transcription factor will unbind (at rate *β*_1_), and the gene will become inactive again. Each copy of mRNA produced will also be degraded (at rate *β*_2_).

In the so called “standard model” of gene expression, Figure 1, an inactive gene can be activated by a promoter or transcription factor. This allows molecules of RNA polymerase to bind and produce mRNA. This in turn can bind to ribosomes leading to the production of protein molecules. Eventually the transcription factor unbinds, terminating the production of mRNA, and each mRNA molecule is degraded, which stops protein production.

Each of these processes is modelled as a transition in a continuous time Markov chain with a particular rate. Such a rate is interpreted as the probability of an event occurring in a unit time interval. Thus, if we denote the rate of transcription factor binding by *α*_0_ then the probability of this occurring in an interval of length *δt*, assuming that the transcription factor is not bound at the start of the interval, is *α*_0_ *δt*. Integrating over time, this means that the probability of the event having happened by time *t*, is 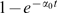, whilst the average time for the event to happen is 1/*α*_0_. The same holds for the other transitions in the model, with the rate of transcription factor unbinding denoted by *β*_1_. Whilst the transcription factor is bound, RNA polymerase binds at a rate *α*_1_, and each such binding event is assumed to produce one molecule of mRNA. More detailed models that allow the polymerase to unbind before it has produced mRNA are considered later and will have no effect on our overall conclusions.

Each mRNA molecule binds to a ribosome at rate *α*_2_ and is degraded at rate *β*_2_. When the last mRNA has decayed no more protein will be produced. We define the number of proteins produced between the transcription factor binding and the last mRNA decaying as a “burst”. Note that since a burst begins once the transcription factor has bound, we expect the distribution of burst sizes to be independent of the transcription factor binding rate *α*_0_. This is confirmed by the rigorous derivation below.

Mathematically, the Standard Model of Gene Expression is a continuous time Markov chain model. Each particular combination of number of mRNA molecules, number of protein molecules and state of binding of the transcription factor constitutes a single state of the model. It is possible to derive an (infinite) set of coupled ordinary differential equations (called the Kolmogorov forward equations or master equation) that govern the probability at any given time of the system being in any given state. However, the analysis of a such a complex set of equations is difficult. On the other hand, using the same approach as for multi-stage queues, it is relatively easy to derive the distribution of protein burst sizes.

### The Component mRNA and Protein Distributions

We begin with the analysis of McAdams and Arkin [3] for the distribution of the number of proteins produced by a single mRNA molecule. If a certain number (possibly 0) of protein molecules has been produced, the probability that the next event in which the mRNA molecule participates is the production of another protein molecule is *p* = *α*_2_/*α*_2_+*β*_2_) (see Text S1 for derivation). Conversely, the probability that the next event is the degradation of the mRNA molecule is 1−*p* = *β*_2_/(*α*_2_+*β*_2_). In order to produce precisely *n* molecules of protein, we need *n* events of the first type to occur, followed by a final degradation event. The probability of this happening is *p^n^*(1−*p*), giving the distribution *Q*(*n*) of the number of protein molecules produced by a single mRNA molecule
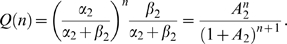
(1)


Here *A*_2_ = *α*_2_/*β*_2_ is the expectation of *Q*. Contrasting this with [3], the parameter *A*_2_ defining the distribution is now expressed in terms of physically measurable rate constants. Exactly the same argument applies to the distribution of the number of RNA molecules produced between the successive binding and unbinding of the transcription factor. In particular, the probability of producing one more mRNA molecule before the transcription factor unbinds is *α*_1_/(*α*_1_+*β*_1_) and the probability of the transcription factor unbinding is *β*_1_/(*α*_1_+*β*_1_). In order to produce precisely *m* mRNA molecules before the transcription factor unbinds we need *m* independent production events with probability *α*_1_/(*α*_1_+*β*_1_), followed by the unbinding event with probability *β*_1_/(*α*_1_+*β*_1_).

Thus the probability distribution, *R*(*m*), of the number of mRNA molecules produced in one burst is
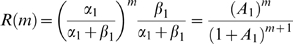
(2)where *A*_1_ = *α*_1_/*β*_1_ is the expectation of *R*(*m*). In order to derive the overall protein burst size distribution for the Standard Model in Figure 1 we need the probability generating functions [17] of the distributions *Q*(*n*) and *R*(*m*) which we denote as Q*(*z*) and R*(*z*), respectively. These are simply obtained by summing the relevant geometric series
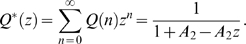
and
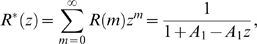



## Results

### The Compound Protein Burst Size Distribution

The distribution *P*(*n*) of the total number of proteins produced in a single burst is simply the compound distribution of *R* and *Q* [17]. This is easily computed using probability generating functions (see below), and is not a geometric distribution. However, it is of relatively little interest since it includes the possibility that the transcription factor unbinds before any proteins have been produced (either because no mRNA is produced, or because this mRNA is degraded before binding to a ribosome). Such events cannot be observed in the experimental protocol used in [6],[7], and hence *P*(*n*) cannot be directly compared to the data in these papers. However, we can re-scale *P*(*n*) to give the probability distribution *Pˆ*(*n*) = *P*(*n*)/(1−*P*(0)) of protein numbers conditional on at least one protein being produced. An approximate calculation of this distribution was given in the supplementary material of [7]. This replaced the discrete geometric distribution *Q*(*n*) by a continuous exponential distribution of the same mean and then used the Laplace transform to obtain the (continuous approximation to the) compound distribution. Here we present an exact derivation for the discrete distribution using generating functions (which are closely related to the Laplace transform). Furthermore we relate the parameter of the final burst size distribution to the original kinetic parameters *α*_1_, *α*_2_, *β*_1_, and *β*_2_.

Thus, let *X* ^(*i*)^ be the random variable, with distribution *Q*(*n*), giving the number of proteins produced by the *i*th mRNA transcript and let *Y* be a random variable, with distribution *R*(*n*) giving the number of mRNA molecules produced. Then the random variable
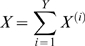
gives the total number of proteins in a burst. Denote the distribution of *X* by *P*(*n*), with generating function P*(*z*). Then a standard result on generating functions of compound distributions [17] gives
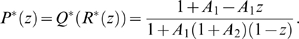
(3)To obtain the distribution conditional on at least one protein molecule being produced, we subtract P*(0) and normalise (divide) by 1−P*(0) to give
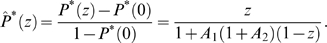
This is the generating function of a conditional geometric distribution with (dimensionless) parameter *Â*_2_ = *A*_2_(1+*A*_1_), so that *Pˆ*(*n*) has the distribution
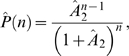
(4)where the parameter *Â*_2_ can be expressed in terms of the mean number *A*_1_ of mRNA molecules produced and the mean number *A*_2_ of protein molecules produced from a single mRNA molecule as
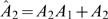
(5)

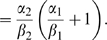
(6)We thus see that the burst size distribution is determined by a single parameter, and that many different combinations of the parameters *α*_1_, *α*_2_, *β*_1_, and *β*_2_ will lead to the same burst size distribution. In mathematical language this says that the Standard Model with parameters *α*_1_, *α*_2_, *β*_1_, and *β*_2_ is *nonidentifiable* from burst size data. In fact we can only estimate a single parameter (or a single linear combination) and the three remaining parameters can be arbitrarily chosen.

### Burst Distributions for Extensions of the Standard Model

It might be hoped that such nonidentifiability is a particular pathology of the Standard Model. We thus next consider a number of generalisations of this model, which provide a more detailed description of the process of gene expression. We find that for a wide range of generalisations we can still derive the burst size distribution in a similar manner the above. It turns out to be geometric in each case and hence all such models are also nonidentifiable.

**Figure 2:**
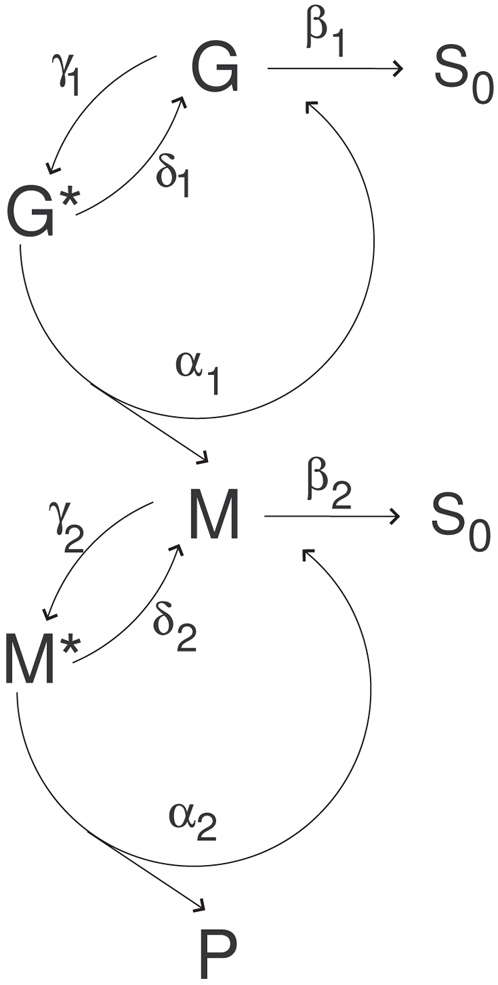
Diagram of the generalised situation in which intermediate, reversible stages are introduced. Here, G represents an active gene, G* an active gene with a bound RNA polymerase, M an mRNA molecule, M* an mRNA molecule bound to a ribosome, P a protein, and *S*_0_ states which correspond to transcription factor unbinding and mRNA transcript decay.

One common extension is to include an additional step in the model of the transcription process [13], as shown in Figure 2. This accounts for the fact that after the transcription factor has bound, one still requires the RNA polymerase to bind to the transcription initiation complex, and this may not always happen successfully. A similar modification could be made to the translation loop to describe the binding of the mRNA transcript to the ribosome in more detail. Both of these additions can be considered individually, or in combination.

Doing this results in distributions *R* and *Q* which are still geometric, but with the parameters *A*_1_ and *A*_2_ given by more complex combinations of the individual rates. We illustrate this for the transcription loop, where we find that in order to produce exactly *m* mRNA molecules, the system can pass through state G* any number *i*≥*m* times. On *i*−*m* of these occasions the polymerase unbinds before an mRNA molecule is produced, returning to *G* with rate *δ*_1_, and on the remaining *m* occasions an mRNA molecule is produced, with rate *α*_1_. The *m* productive steps can be interspersed in any order amongst the *i* visits, giving 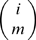 possible choices. The probability of producing *m* mRNA molecules is thus
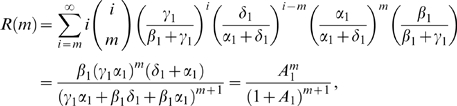
with *A*_1_ now given by *A*_1_ = *α*_1_ *γ*_1_/*β*_1_(*α*_1_+*δ*_1_). A similar derivation holds for the translation loop. We see that carrying out either or both of these modifications still results in a geometric distribution in the form of Equation 4 for *Pˆ*(*n*), with *Â*_2_ = *A*_2_(1+*A*_1_), but *A*_1_ and *A*_2_ now given by *A*_1_ = *α*_1_ *γ*_1_/*β*_1_(*α*_1_+*δ*_1_) and *A*_2_ = *α*_2_ *γ*_2_/*β*_2_(*α*_2_+*δ*_2_). As a consequence the overall conditional protein size distribution, *Pˆ*(*n*), will still be given by Equation 4, with the parameter *Â*_2_ = *A*_2_ *A*_1_+*A*_2_ as before.

An alternative generalisation is to add additional loops with the same structure as the current transcription and translation loops. We prove in the Supporting Information (Text S1) that if we have *k*−1 such loops, the final conditional protein size distribution *Pˆ* *_k_*(*n*) will still be geometric.

We thus conclude that all of these models yield the same geometric protein burst size conditional distribution, determined by a single parameter. In particular, models which include additional steps to account for DNA–RNAP complex formation and mRNA-ribosome complex formation give distributions that are mathematically indistinguishable from those from the Standard Model. It is thus impossible to differentiate between these models using experimentally observed burst size distributions. Similarly we cannot use such data to differentiate between the contributions to noisy gene expression from transcriptional versus translational bursting.

### Comparison with Burst Size Data

We can compare the probability distribution derived above directly with experimental data. We consider recently published data of burst sizes for two fluorescently tagged proteins in the bacterium *Escherichia coli* [6],[7]. In [6], a novel fluorescent imaging technique is used to determine the distribution of protein molecules per transcription factor binding event in live *E. coli* cells. The specific protein studied was a fusion of a yellow fluorescent protein variant (Venus) with the membrane protein Tsr. The *tsr-venus* gene is incorporated into the *E. coli* chromosome, replacing the *lacZ* gene. This modified gene is then under the control of the *lac* promoter. In a second publication [7], the same group used a different imaging technique to determine the distribution of protein molecules per transcription factor binding event of *β*-gal in live *E. coli* cells.

**Figure 3:**
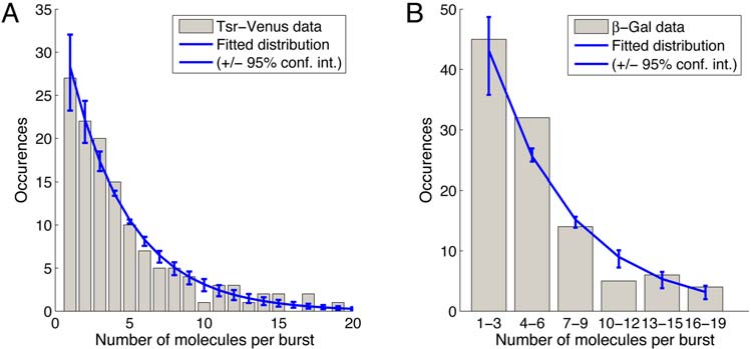
Comparison of the distribution of experimentally measured burst sizes for the proteins *Tsr-Venus* (A) [6] and for the *β-gal* (B) [7] with the standard model of gene expression. In both cases the blue line shows the best fit of the model to the data, obtained using the method of maximum likelihood giving *Â*_2_ = 3.57 for *Tsr-Venus* and *Â*_2_ = 20.96 for *β-gal*. The error bars show the upper and lower bounds of the 95% confidence interval for the fitted parameter.

Such experimental data can be compared to the predicted distribution *Pˆ*(*n*) in two ways. One possibility is to use maximum likelihood estimation to find the value of *Â*_2_ for which *Pˆ*(*n*) best fits the data. This is illustrated in Figure 3, which shows that it is possible to obtain excellent agreement between the theoretical and experimental distributions. The estimated value of *Â*_2_ for Tsr-Venus is *Â*_2_ = 3.57, whilst for *β*-gal, *Â*_2_ = 20.96. The difference in magnitude between these two estimates may be partially due to the fact that *β*-gal is only active as a tetramer. Thus, each burst of activation measured experimentally (and thus available for fitting) corresponds to the production of 4 monomers. The disadvantage of fitting the model in this way is it can only provide an estimate of the single parameter *Â*_2_, but not of the underlying kinetic parameters *α*_1_, *α*_2_, *β*_1_, and *β*_2_.

An alternative approach to verifying the model would be to obtain independent estimates of the model parameters from which we can calculate *Â*_2_ using Equation 6. The resulting geometric distribution can then be compared to the observed burst size data. Unfortunately, as is common for most models in cell and molecular biology, direct experimental measurements of many of these rates are not available. For the *β*-gal data, *β*_2_ can be obtained from the reported mRNA half life [7],[20], but the other three parameters corresponding to the off-rate of the transcription factor and to the binding rates of RNA polymerase to DNA and of mRNA to ribosome respectively are not available.

### Application to Experimental Data

#### Incorporating steady state distribution data

**Figure 4:**
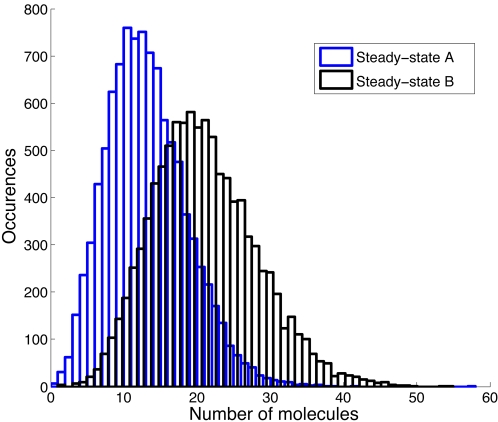
Simulations of steady-state protein expression levels. For A we have *α*_1_ = 0.018 and *β*_1_ = 0.086 and for B we have *α*_1_ = 0.009 and *β*_1_ = 0.043, resulting in the same *Â*_2_ and hence identical burst size distributions. Other parameters were *α*_0_ = 0.012, *α*_2_ = 0.013, *β*_2_ = 0.0039, and *β*_3_ = 0.0007, based on previous simulation studies [22]. The distributions shown are for a run of 10,000 seconds using the Stocks implementation of Gillespie's method [23], after an initial transient of 10,000 seconds. Previous studies have indicated that the steady state is in fact attained in under 1000 seconds.

We thus conclude that we can neither estimate all the kinetic parameters *α*_1_, *α*_2_, *β*_1_, and *β*_2_ from the burst size data, nor measure them by other means. However, experience suggests that by supplementing the burst size distribution with other experimental data it may be possible to overcome the nonidentifiability of these parameters. This is reinforced by the observation that parameter combinations that lead to the same *Â*_2_ and hence the same burst size distribution can yield quite different steady-state distributions, as shown for example in Figure 4. The two steady state distributions shown have different choices of *α*_1_, *α*_2_, *β*_1_, and *β*_2_ that yield the same value for *Â*_2_, and hence the same burst size distribution. However, the two steady state distributions are clearly different. This shows that steady state distribution data should allow us to distinguish between different combinations of parameters with the same *Â*_2_, and hence potentially identify some or all of these parameters.

#### Empirical likelihood estimation

The main difficulty with such an approach is the lack of analytic expressions for the steady state distribution, making it impossible to derive an explicit formula for the likelihood. Instead one has to compute an estimate of the equilibrium distribution using simulations of the reaction network [20] and then use these to derive an empirical likelihood by comparing to the experimental data. This can then be maximized in the usual way.

We applied this approach to the data from [7], which presents both burst size and steady state distributions for the same experimental system. In order to fully specify the steady state distribution, we need two additional parameters: the rate of transcription factor binding *α*_0_ and the rate of protein decay *β*_3_. These do not enter into the expressions for the burst size distribution, and were assumed to be known (and fixed) for the simulations shown in Figure 4. In the absence of independent estimates of these parameters for the *β*-gal system, we explored the possibility of estimating these from the data in [7] directly by computing an empirical likelihood using simulation of the model (see below). We attempted both to maximize this empirical likelihood directly, and to obtain its distribution using Markov Chain Monte Carlo sampling. Neither of these approaches were successful with the full six parameter model (results not shown).

We can however, make use of independent estimates of parameters in the model to reduce the dimensionality of the parameter space. In effect this constrains the orginal optimization to a lower dimensional sub-space. We applied this approach with two different choices of parameter: the mRNA degradation rate *β*_2_ and the protein degradation rate *β*_3_.

#### Constraining on the mRNA degradation rate

We chose first to make use of the wide availability of estimates of the value of *β*_2_, the rate of mRNA degradation. Since we also have the burst size data, we first estimate *Â*_2_ and then use Equation 6 to obtain an expression for *α*_2_ in terms of *α*_1_, *β*_1_, and *β*_2_. We are left with the four dimensional parameter space *α*_0_, *α*_1_, *β*_1_, and *β*_3_. At each point in this space, we simulate the model using the Gillespie algorithm to given an empirical estimate of the probability *P_n_*(*α*_0_,*α*_1_,*β*_1_,*β*_3_) of observing *n* proteins at equilibrium. This gives the empirical log-likelihood
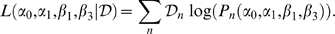
where 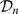 is the number of times that *n* proteins are observed in the experimentally data 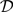.

**Figure 5:**
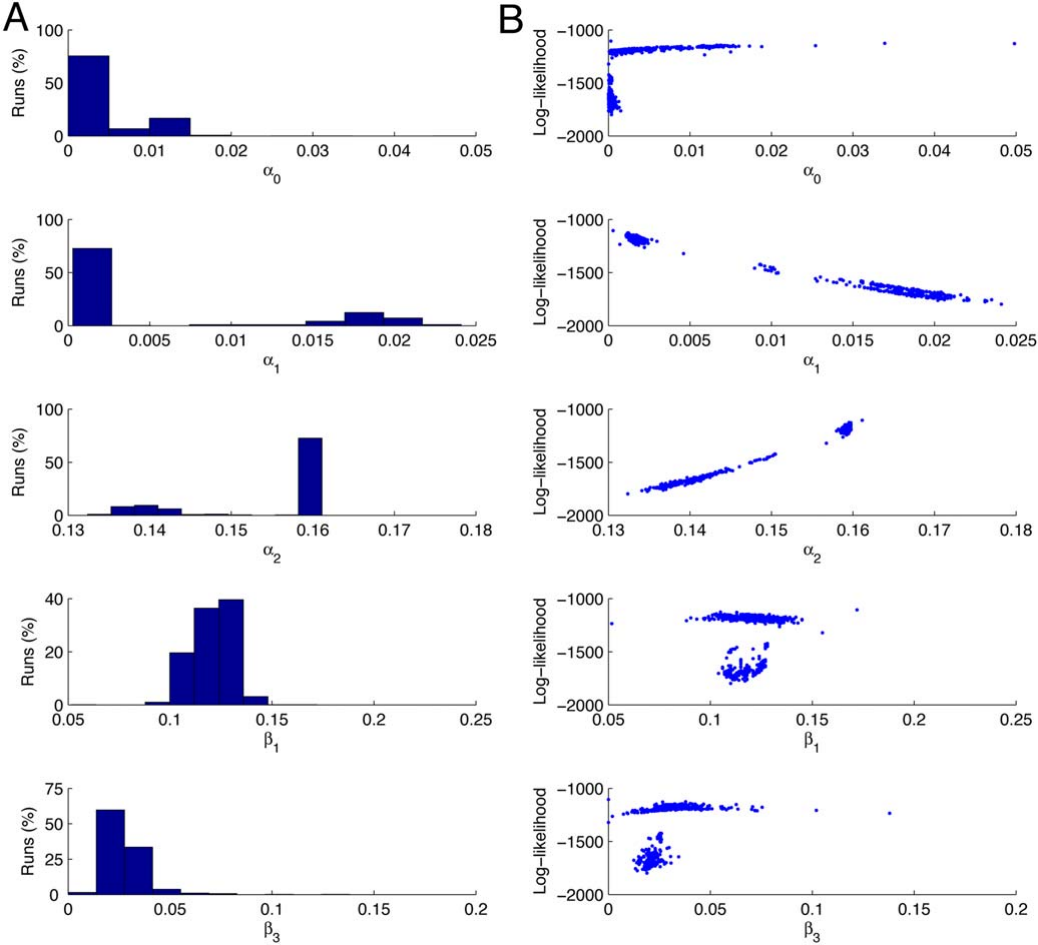
Parameter estimation results with fixed mRNA degradation rate. The results of 1000 runs of the Nelder-Mead maximisation of the log-likelihood for the parameters *α*_0_, *α*_1_, *β*_1_, and *β*_3_, with *α*_2_ determined by the relationship in Equation 6, and with the mRNA degradation rate *β*_2_ set to 7.2×10^−3^, corresponding to a half-life for *β*-gal mRNA of 1.6 mins [24]. The panels in column A show the estimates of the values of the parameters and the percentage of times the Nelder-Mead algorithm converged to those values. The panels in column B are scattergrams of the values of the parameter estimates against the value of the log-likelihood. Each simulation is run 10,000 times to simulate a population of 10,000 cells, and each simulation is run for 5000 reaction steps. The starting values for the optimisation routine are: *α*_0_ = 0.01 s^−1^, *α*_1_ = 0.02 s^−1^, *β*_1_ = 0.1 s^−1^, and *β*_3_ = 0.0007 s^−1^, and are based on previous simulation studies [16].

This empirical likelihood function can be maximized using any suitable optimization algorithm. Because it is computed using stochastic simulations any particular realization of the function is not smooth, making algoritms that use gradient (or Hessian) information unsuitable. We therefore opted to use the Nelder-Mead (simplex) method [21] with the results shown in Figure 5.

We can see that the likelihood has a local maximum at *L*≈−1800, and the simplex method frequently gets stuck in this region. However the majority of runs (73.4%) converge to the presumed global maximum. The means and standard deviations of the estimated parameter values are shown in Table 1. The value obtained for *β*_3_ is 0.0297 s^−1^, which corresponds to a half-life of 23 seconds. This appears to be unrealistically short, since *β*-galactosidase is a stable protein with a reported lifetime of hours.

**Table 1:** Means and standard deviations of estimates of the other parameters when the mRNA degradation rate *β*_2_ set to 7.2×10^−3^.

	*α*_0_	*α*_1_	*α*_2_	*β*_1_	*β*_3_
*μ*	0.0049	0.0017	0.1538	0.1210 (*τ*_1/2_ = 5.7 s)	0.0297 (*τ*_1/2_ = 23.3 s)
*σ*	0.0052	0.0003	0.0088	0.0098	0.0098

These statistics are based on those runs that approached the global maximum (73.4% of all runs). These were selected by imposing a threshold at *L* = −1350 and only considering those runs converging to a larger (more positive) likelihood. All reaction rates have units of s^−1^.

One possible explanation of this discrepancy is that the reported mRNA degradation rate *β*_2_ = 7.2×10^−3^ is always measured in experimental conditions where the gene is active. On the other hand, the burst size and equilibrium distributions in [7] are obtained under conditions where the gene is suppressed. It is possible that the mRNA degradation rates are significantly different in the two cases. To explore this hypothesis, we approached the problem from an alternative direction, fixing the protein degradation rate *β*_3_ to correspond to a half-life of 1 hour, and estimating the remaining five parameters, including mRNA degradation rate *β*_2_.

#### Constraining on the protein degradation rate

**Figure 6:**
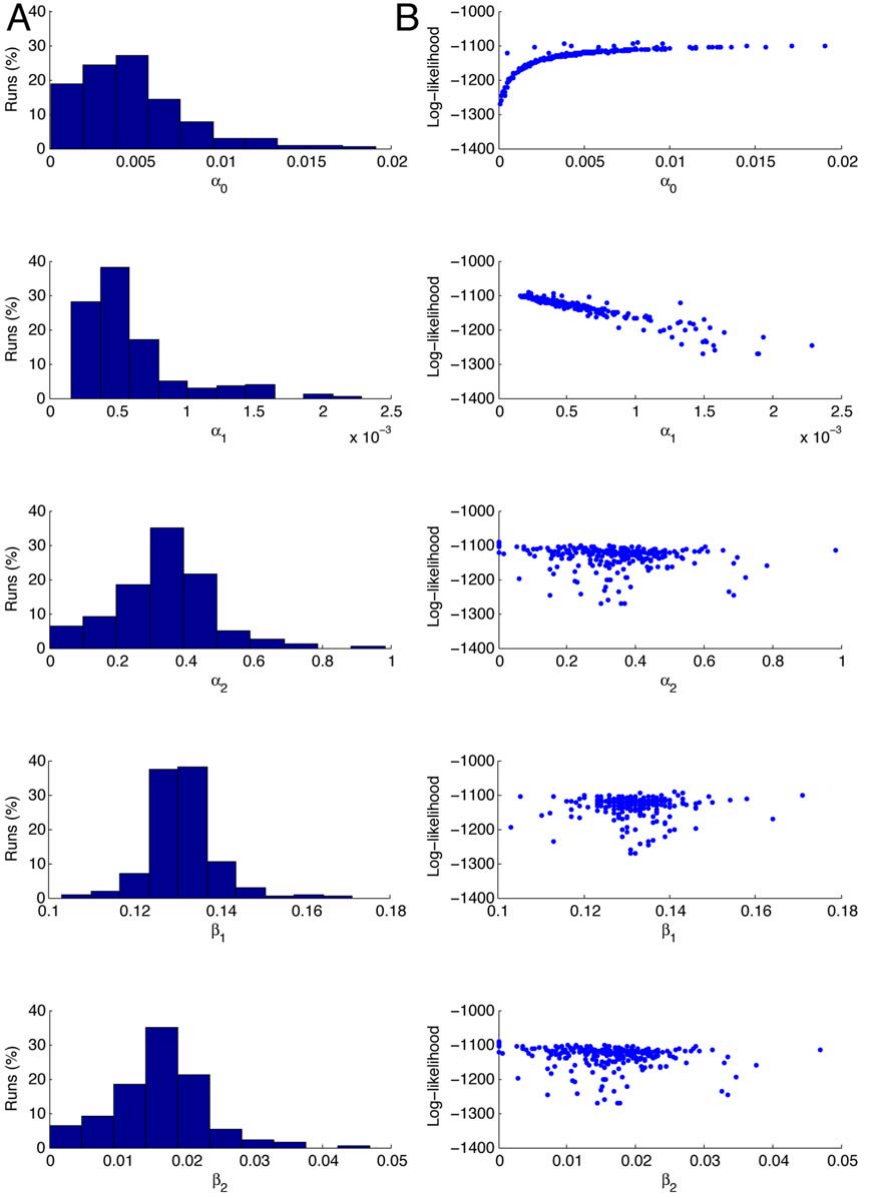
Parameter estimation results with fixed protein degradation rate. The results of 10,000 runs of the Nelder-Mead maximisation of the log-likelihood for the parameters *α*_0_, *α*_1_, *β*_1_, and *β*_2_, with *α*_2_ determined by the relationship in Equation 6, and with the protein degradation rate set, *β*_3_ set to 2.77×10^−4^, consistent with a half-life for *β*-gal 60 mins. The panels in column A show the estimates of the values of the parameters and the percentage of times the Nelder-Mead algorithm converged to those values. The panels in column B are scattergrams of the values of the parameter estimates against the value of the log-likelihood. Each simulation is run 10,000 times to simulate a population of 10,000 cells, and each simulation is run for 5000 reaction steps. The starting values for the optimisation routine are: *α*_0_ = 0.01 s^−1^, *α*_1_ = 0.02 s^−1^, *β*_1_ = 0.1 s^−1^, and *β*_2_ = 0.007 s^−1^, and are based on previous simulation studies [16].

We therefore fixed *β*_3_ to 1.92×10^−4^ s^−1^, corresponding to a protein half-life of one hour, and then used same method as described above to estimate the other parameters *α*_0_, *α*_1_, *α*_2_, *β*_1_, and *β*_2_. We ran 10,000 simulations, as a relatively low number of runs converged (23.37%), with the others becoming trapped in a region with physically unrealistic (negative) reaction rates, and a log-likelihood of *L*≈−2100. Of the runs which converged, 2057 (88%) converged to a local maximum at *L*≈−9150, while 279 (12%) converged to the presumed global maximum at *L*≈−1100. The results for the runs which converged can be seen in Figure 6, whilst summary statistics for the runs which converged to the presumed global maximum are presented in Table 2.

**Table 2:** Means and standard deviations of the parameter estimates, when the protein degradation rate *β*_3_ set to 1.92×10^−4^ (*τ*_1/2_ = 3600 s).

	*α*_0_	*α*_1_	*α*_2_	*β*_1_	*β*_2_
*μ*	0.0048	0.0006	0.3352	0.1314 (*τ*_1/2_ = 5.3 s)	0.0161 (*τ*_1/2_ = 43.1 s)
*σ*	0.0033	0.0004	0.1476	0.0078	0.0071

These statistics are based on those runs which approached the global maximum (12% of converging runs). These were selected by imposing a threshold at *L* = −2500 and only considering those runs converging to a larger (more positive) likelihood. All reaction rates have units of s^−1^.

#### Comparison of the estimates

The transcription factor binding rate *α*_0_ is almost unchanged under both assumptions. When we fix the protein degradation rate in the second set of estimates to a value much lower than estimated in the first set we find that the transcription rate (i.e., rate of RNA polymerase binding) *α*_1_, is approximately one third of the previous value, decreasing from 0.0017 s^−1^ to 0.0006 s^−1^, whilst the translation rate, *α*_2_ shows an approximate two-fold increase, from 0.1538 s^−1^ to 0.3352 s^−1^. It is intuitively reasonable that such a combination of decreasing transcription and increasing translation leads to the same overall level of protein expression. The parameter *β*_1_, the rate at which the transcription factor unbinds increases slightly, leading to shorter bursts. The increase in the mRNA degradation rate, *β*_2_ from the original assumption of 0.007 s^−1^ to the estimate of 0.0161 s^−1^ (corresponding to an mRNA half life of approximately 43 seconds) suggests that when expression of the gene is being strongly repressed as in this situation, there may well be active degradation of the mRNA. It would be interesting to experimentally investigate this biologically significant prediction.

## Discussion

We have shown that it is possible to use results from queuing theory to derive the burst size distribution of protein molecules produced by a single transcription factor binding event in terms of physically measurable kinetic rate constants for both the simplest model of gene expression, the so-called Standard Model, and for a number of natural extensions.

Furthermore, we have shown that the mathematical form of these models is nonidentifiable, and all such burst size distributions are actually determined by a single parameter. This implies that it is impossible to use burst size data alone to determine the relative contributions of transcription and translation to the variability in gene expression.

One possible way of overcoming this limitation is to use a combination of burst size data and steady-state data. However, this requires estimates of a further two parameters (which are not needed when using burst-size data alone). We were unable to estimate all six parameters directly from the combined data. However, using independent estimates of either the mRNA lifetime or the protein lifetime reduces the number of parameters by one, and enables successfully estimation of the remaining five parameters by maximizing an empirical likelihood using the Nelder-Mead simplex algorithm. Although this suffers from the common problem of occasional convergence to a local maximum, by using computing repeated estimates it was possible to identify and exclude such cases and hence obtain good estimates of the desired five kinetic parameters under the different constraints.

## Supporting Information

Text S1 Derivation of probabilities.
(0.10 MB PDF)
