# Supplementary material for: Nonidentifiability of the Source of Intrinsic Noise in Gene Expression from Single-Burst Data
Source: PLoS Comput Biol. 2008 Oct 10;4(10):e1000192. doi: 10.1371/journal.pcbi.1000192 (PMC2538572; doi:10.1371/journal.pcbi.1000192)
Supplement: Text S1 — Derivation of probabilities. (0.10 MB PDF) [file pcbi.1000192.s001.pdf]

Non-identifiability of the Source of Intrinsic Noise in Gene Expression From Single-Burst Data - Supplementary Material

Piers J. Ingram[1,2,3], Michael P.H. Stumpf[2,3], Jaroslav Stark[1,2]

15th August 2008

Derivation of Probabilities

Consider a step in a Markov chain, as in Figure S1. We suppose that at time t_0 the system is in state 0 from which it can make two possible transitions, to either state 1 or state 2, with rates α and β respectively. The probability that it is still in state 0 at some time $t > t_0$ is $e^{-(\alpha+\beta)(t-t_0)}$ and therefore the system is certain to eventually move to either state 1 or state 2. We wish to compute the probabilities of these two possibilities, irrespective of when they happen. The probability that the transition occurs between t and $t+\delta t$ is $(\alpha+\beta)\delta t\, e^{-(\alpha+\beta)(t-t_0)}$. The probability that the transition during this time is to state 1 is $\alpha\,\delta t\, e^{-(\alpha+\beta)(t-t_0)}$ and the probability that it is to state 2 is $\alpha\,\delta t\, e^{-(\alpha+\beta)(t-t_0)}$. Hence the probability that the the next state is 1 is

$$p = \frac{\alpha\,\delta t\, e^{-(\alpha+\beta)(t-t_0)}}{(\alpha+\beta)\delta t\, e^{-(\alpha+\beta)(t-t_0)}} = \frac{\alpha}{\alpha+\beta},$$

and the probability that the next state is 2 is

$$1 - p = \frac{\beta}{\alpha+\beta}.$$

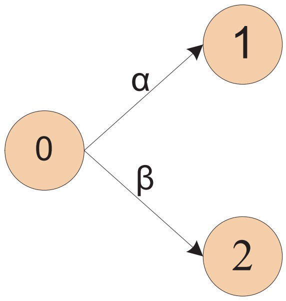

Figure S1: If the system is in state 0 at a given time, it can transit to state 1 at a rate α or to state 2 at a rate β. The probability that the system will transit from state 0 to step 1 in an arbitrary time-step h is αh.

[1]Department of Mathematics, Imperial College London, London, SW7 2AZ, UK.
[2]Centre for Integrative Systems Biology at Imperial College (CISBIC), Imperial College London, London, SW7 2AZ, UK.
[3]Theoretical Genomics Group, Centre for Bioinformatics, Division of Molecular Biosciences, Imperial College London, London, SW7 2AZ, UK.

The Joint Distribution In the main paper we have given the overall protein burst size distribution $P(n)$. It is also possible to derive the more detailed joint distribution $P(m, n)$ that exactly m mRNA and n protein molecules are produced. We may think of this as

$$P(m, n) = P(n|M = m)R(m),$$

where $P(n|M = m)$ is the conditional distribution that n proteins are produced if there are m mRNA molecules. If we assume that each transcript produces copies of the protein independently then the generating function $P^*(z|m)$ is just the product of the m generating functions for the protein produced by one mRNA molecule,

$$P^*(z|m) = [Q^*(z)]^m = \left(\frac{1}{1 + A_2 - A_2 z}\right)^m .$$

Hence to compute the probabilities $P(n|M = m)$, we calculate

$$\begin{aligned} P(n|M = m) &= \frac{1}{n!}\frac{d^n}{dz^n}\left\{[Q^*(z)]^m\right\}_{z=0} \\ &= \frac{1}{n!}\frac{d^n}{dz^n}\left\{\frac{1}{(1 + A_2 - zA_2)^m}\right\}_{z=0} . \end{aligned}$$

For the case $n = 1$, we may easily compute

$$P(1|M = m) = \frac{1}{(1 + A_2)^{m+1}}.$$

We now prove the more general result using the case $n = 1$ as a basis for induction. Assuming that for the case $n = i$:

$$P(i|M = m) = \frac{(m + i - 1)!}{i!(m - 1)!}\frac{A_2^i}{(1 + A_2 - zA_2)^{m+i}},$$

then for $n = i + 1$:

$$\begin{aligned} \frac{d^{i+1}}{dz^{i+1}}\left\{[Q^*(z)]^m\right\} &= \frac{d}{dz}\frac{d^i}{dz^i}\left\{[Q^*(z)]^m\right\} \\ &= \frac{d}{dz}\left(\frac{(m + i - 1)!}{(m - 1)!}\frac{A_2^i}{(1 + A_2 - zA_2)^{t+i}}\right) \\ &= \frac{(m + i - 1)!(m + i)}{(m - 1)!}\frac{A_2 A_2^i}{(1 + A_2 - zA_2)^{m+i+1}} \\ &= \frac{(m + i)!}{(m - 1)!}\frac{A_2^{i+1}}{(1 + A_2 - zA_2)^{m+i+1}} \end{aligned}$$

which completes the inductive step. Therefore

$$P(n|M = m) = \frac{(m + n - 1)!}{n!(m - 1)!}\frac{A_2^n}{(1 + A_2)^{m+n}}.$$

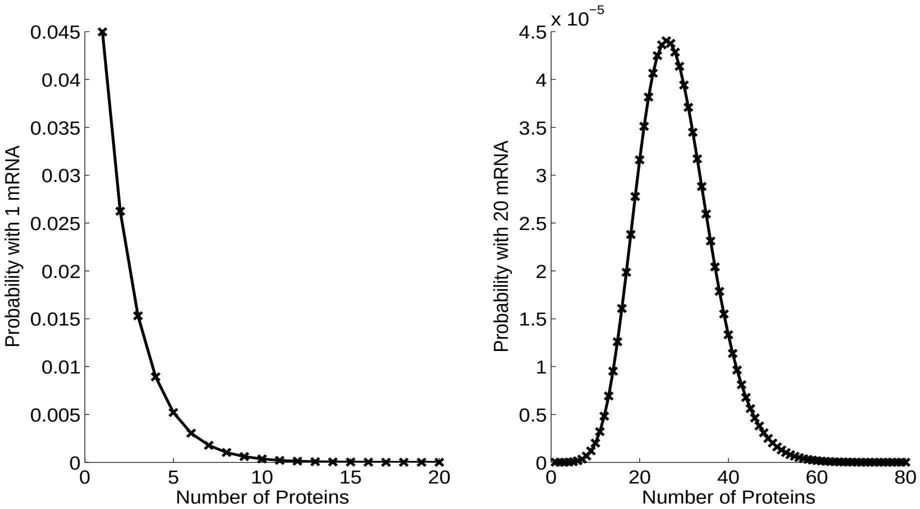

Figure S2: Distribution of the number of proteins which will be produced during a gene expression burst with one mRNA molecule and with twenty mRNA molecules.

Thus the joint probability may now be calculated as

$$\begin{aligned} P(n,m) &= P(n|M=m)R(m) \\ &= \frac{(m+n-1)!}{n!(m-1)!}\frac{A_2^n}{(1+A_2)^{m+n}}\frac{A_1^m}{(1+A_1)^{m+1}}. \end{aligned}$$

This is illustrated for two different values of number of mRNA molecules in Figure S2.

Finally, by summing over m we can recover the overall burst size distribution $P(n)$ which was derived using generating functions (but only the conditional distribution for $n > 0$ was explicitly stated). Special consideration is needed for the case $n = 0$, as the case that no transcripts are produced must be added to the probability that m transcripts are produced but no proteins are produced. Thus

$$P(0) = \frac{A_1}{1+A_1}\frac{1}{(1+A_2+A_1A_2)} + \frac{1}{1+A_1},$$

and for $n > 0$

$$P(n) = \sum_{m=1}^{\infty} P(n,m) = \frac{A_1}{1+A_1}\frac{(A_2+A_1A_2)^n}{(1+A_2+A_1A_2)^{n+1}}.$$

Conditioning on $n > 0$ and defining $A_2 = A_2(1 + A_1)$ recovers $\hat{P}(n)$ as in the main article. Similar calculations can be carried out for the various extensions to the standard model considered above, though the details become quite lengthy for the more complex cases.

Alternative generalisation

A different generalisation is to add additional loops with the same structure as the current transcription and translation loops, Figure S3. We prove below that if we have $k-1$ such loops,

the final conditional protein size distribution $\hat{P}_k(n)$ will still be geometric

$$\hat{P}_k(n) = \frac{\hat{A}_k^{n-1}}{(1+\hat{A}_k)^n}, \tag{S1}$$

with the parameter $\hat{A}_k$ given by

$$\hat{A}_k = A_k + A_k A_{k-1} + .. + A_k A_{k-1} ... A_1 = \sum_{i=1}^{k} \prod_{j=i}^{k} A_j. \tag{S2}$$

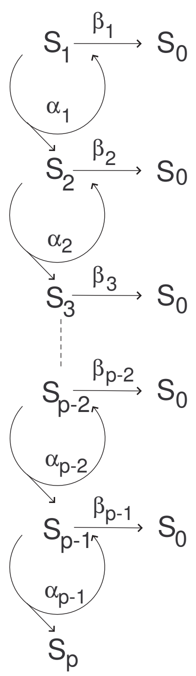

Figure S3: Diagram of the generalised situation with $k-1$ serially coupled loops of the type considered. If $k = 3$ then we have a system with two loops which we have used to model transcription and translation in gene expression.

By induction, suppose that at the kth stage the conditional distribution $\hat{P}_k(n)$ is geometric and has generating function $\hat{P}_k^*(z) = z/(1+\hat{A}_k(1-z))$. If the generating function for the next loop is $Q_{k+1}^*(z) = 1/(1+A_{k+1}(1-z))$ then adding this loop gives $\hat{P}_{k+1}^*(z) = Q_{k+1}^*(\hat{P}_k^*(z)) = (1+\hat{A}_k(1-z))/(1+A_{k+1}(1+\hat{A}_k)(1-z))$. This has the same form as $\hat{P}^*(z)$ given in the main text, and so carrying out the conditioning on $n > 0$ gives $\hat{P}_k^*(z) = z/(1+A_{k+1}(1+\hat{A}_k)(1-z))$ completing the inductive step with $\hat{A}_{k+1} = A_{k+1}(1+\hat{A}_k)$.

Iterating this with initial condition $\hat{A}_1 = A_1$ gives the expression in Equation S2.
